# Diabetes Mellitus and Heart Failure: Epidemiology, Pathophysiologic Mechanisms, and the Role of SGLT2 Inhibitors

**DOI:** 10.3390/life13020497

**Published:** 2023-02-10

**Authors:** Panagiotis Theofilis, Evangelos Oikonomou, Konstantinos Tsioufis, Dimitris Tousoulis

**Affiliations:** 1 Department of Cardiology, “Hippokration” General Hospital, University of Athens Medical School, 11527 Athens, Greece; 2 Department of Cardiology, Thoracic Diseases General Hospital “Sotiria”, University of Athens Medical School, 11527 Athens, Greece

**Keywords:** heart failure, diabetes mellitus, SGLT2 inhibitors

## Abstract

Diabetes mellitus (DM) and heart failure (HF) are frequently encountered afflictions that are linked by a common pathophysiologic background. According to landmark studies, those conditions frequently coexist, and this interaction represents a poor prognostic indicator. Based on mechanistic studies, HF can be propagated by multiple pathophysiologic pathways, such as inflammation, oxidative stress, endothelial dysfunction, fibrosis, cardiac autonomic neuropathy, and alterations in substrate utilization. In this regard, DM may augment myocardial inflammation, fibrosis, autonomic dysfunction, and lipotoxicity. As the interaction between DM and HF appears critical, the new cornerstone in DM and HF treatment, sodium-glucose cotransporter-2 inhibitors (SGLT2i), may be able to revert the pathophysiology of those conditions and lead to beneficial HF outcomes. In this review, we aim to highlight the deleterious pathophysiologic interaction between DM and HF, as well as demonstrate the beneficial role of SGLT2i in this field.

## 1. Introduction

Diabetes mellitus (DM) is a developing pandemic, with an anticipated global prevalence of 9.3%, and it is estimated that 50% of persons with DM are currently undiagnosed [1]. Due to the nature of its vascular consequences, it is a public health concern precipitating significant morbidity and death [2]. Cardiovascular complications continue to be the greatest cause of morbidity and death among people with DM. Overt cardiovascular diseases, such as obstructive coronary artery disease (CAD), acute ischemic stroke, or critical limb ischemia, are the most prevalent concerns in diabetic individuals [3]. DM is also a major cause of chronic kidney disease, retinopathy, and neuropathy.

DM may be involved in the development and progression of heart failure (HF), which is frequently termed diabetic cardiomyopathy. HF, irrespective of the cause, is characterized by a poor short- and long-term prognosis, with significantly impaired quality of life for the affected individuals. As the connection between DM and HF still remains incompletely understood, in this review article, we aim to provide an overview of the most recent evidence in the epidemiology and pathophysiology as well as the role of sodium-glucose cotransporter-2 inhibitors (SGLT2i) in the management of this deleterious interaction.

## 2. Epidemiological Trends: Heart Failure and Diabetes Mellitus

Since DM is a major risk factor for HF, it is no surprise that the prevalence and incidence rates of HF in individuals with DM are high. The authors of a recent study of 3.25 million individuals found that the presence of DM was associated with two-fold higher HF incidence [4]. We should note that in recent years, the proportion of patients who have suffered from HF that are obese or have DM increased to a large extent, as pointed by the study of Ciardullo et al. [5]. The longer duration and poorer control of DM are factors that signify an augmented risk of HF [4]. Nevertheless, HF may also develop in young individuals with a recent onset of DM [6,7]. A recent diagnosis of type 2 DM or insulin resistance was also associated with a greater risk of incident HF among UKPDS participants [8]. These results confirm previous longitudinal studies supporting such associations [9,10,11]. The increased HF risk may be even greater among women and those with type 1 DM, as shown in a recent systematic review and meta-analysis [12]. It should also be stated that HF with reduced (HFrEF) and preserved (HFpEF) left ventricular ejection fractions (LVEF) are equally common in individuals with DM [13], but HFpEF may remain undetected in a considerable proportion of patients [14].

HF may be the first cardiovascular manifestation of DM, especially among black women, as shown by a recent study conducted by Sinha et al. incorporating 40,117 individuals [15]. The analysis of larger cohorts has also demonstrated that HF is a common initial presentation in patients with DM [16,17]. Several noninvasive risk scores have been proposed with the objective of the risk stratification of patients with DM concerning the risk of incident HF [18]. These include the TRS-HFDM, WATCH-DM, Qdiabetes, BRAVO risk engine, Health ABC HF Score, and RECODe, among others [18]. It is particularly important to note that the co-presentation of DM in patients with HF may predispose them to a worse prognosis. DM was an independent predictor of short-term readmission in a study of 1727 Chinese patients with HF [19]. DM has been previously established as a severe risk factor for mortality in patients with HF [20]. The measurement of N-terminal, pro-B natriuretic peptides may be of importance in the risk prognostication of patients with DM and HF [21], as also endorsed by the latest HF guidelines [22].

The relationship between these two entities is bidirectional, since the development of insulin resistance or DM is common among individuals with HF. However, the reported prevalence of DM among patients with HF varies across studies. Pathological glucose homeostasis prevails in over 20% of patients with HF, with higher rates in clinical trials of pharmacological interventions and acute HF [23]. A contemporary registry of 7488 HF patients reported a DM prevalence of 29% [24]. Ethnic disparities also persist, with lower rates of DM being reported among African HF patients and higher rates among Middle Eastern individuals [25]. In a multinational registry, DM prevalence was 31% [26]. 

It becomes apparent that this interplay between DM and HF is based on shared pathophysiologic mechanisms that influence the natural history of both conditions, which will be discussed below.

## 3. Major Mechanisms in Heart Failure Pathophysiology

### 3.1. Inflammation

Inflammation represents a critical component of the pathophysiology of cardiovascular diseases [27,28,29]. In the setting of HF and irrespective of etiology, the injured myocardium results in the activation of the innate immune system through the interaction of pathogen- or damage-associated molecular patterns with pattern recognition receptors (toll-like receptors (TLRs) and NOD-like receptors), which are located on the myocardium and immune cells and are overexpressed in HF [30]. The ensuing increased excretion of pro-inflammatory cytokines and chemokines leads to endothelial cell activation as well as the activation of adaptive immunity through B and T cells, while the mobilization of immune cells (neutrophils, monocytes, and macrophages) further augments cytokine production and phagocytosis [30]. The association of inflammation with HF has also been determined in observational studies, since increased levels of inflammatory markers were associated with the development of HF [31]. Moreover, high C-reactive protein (CRP) levels at discharge following an acute HF decompensation event may be related to adverse prognosis and mortality [32].

### 3.2. Oxidative Stress

Along with inflammation, oxidative stress is also implicated in the development and progression of HF. The imbalance of the myocardial redox state, including the overproduction of reactive oxygen species (ROS) and defective innate antioxidant mechanisms, can result in adverse cardiac remodeling. Several hazardous effects have been described as a result of excessive ROS production, including cardiomyocyte electrophysiological dysfunction, impaired myocardial contractility due to calcium overload, mitochondrial dysfunction, and fibrosis due to an imbalance between the expression of the tissue inhibitors of metalloproteinases and matrix metalloproteinases. Furthermore, the major antioxidant mechanisms are impaired among individuals with HF, including superoxide dismutase [33], glutathione [34], and nicotinamide adenine dinucleotide [35]. 

### 3.3. Endothelial Dysfunction

Endothelial dysfunction is an additional consideration in the pathophysiology of HF, as it is a sequela of oxidative stress and inflammation [36]. The imbalance between nitric oxide (NO) and superoxide as the principal agents of endothelium-dependent vasodilation is highly prevalent in numerous cardiovascular risk factors (DM, arterial hypertension, and chronic kidney disease) apart from overt CAD, which is a fact that can explain the presence of endothelial dysfunction irrespective of LVEF and HF etiology [37]. Markers of HF and endothelial dysfunction have been correlated in previous studies, indicating the interaction between those two conditions [38]. It should also be noted that endothelial dysfunction is an important prognostic factor in chronic HF, as it is associated with a higher incidence of hospitalization and mortality [39].

### 3.4. Fibrosis

The increased concentration of collagen in the setting of myocardial fibrosis is an essential factor implicated in adverse myocardial remodeling and, eventually, HF development. Apoptotic cardiomyocyte cell death, as seen in acute ischemic events, represents the main source of replacement fibrosis [40]. Initially, fibroblasts are activated directly through microRNAs and matrix metalloproteinases or indirectly through endothelial and inflammatory cells [41]. Following activation, the proliferation and differentiation of fibroblasts has been observed owing to the action of growth factors (transforming growth factor-beta) or inflammatory cytokines (tumor necrosis factor-alpha), which leads to the formation of myofibroblasts, which, in turn, are responsible for collagen deposition in the extracellular matrix [42]. In the absence of apoptosis, other stimuli such as obesity, smoking, arterial hypertension, dyslipidemia, and DM have been implicated in the fibrotic process [43]. Several markers of fibrosis have been associated with HF, including galectin-3 and serum carboxy-terminal propeptide of procollagen type I [44,45]. The presence of fibrosis may also add important prognostic information in HF regardless of LVEF [46]. Previous studies of fibrosis of a histological (collagen volume fraction) or imaging-based (extracellular volume fraction) nature have suggested a deleterious association of fibrosis with adverse HF outcomes [47,48].

### 3.5. Cardiac Autonomic Neuropathy

Cardiac autonomic neuropathy (CAN) is defined by an imbalance between cardiac sympathetic and parasympathetic activity [49]. Due to the increased production of cardiac catecholamines, the activation of adrenergic receptors, and the activation of the renin-angiotensin-aldosterone system (RAAS), sympathetic activity might aggravate cardiomyopathy in individuals with DM. The longest nerve is the vagus nerve (parasympathetic); it innervates the heart and contains 75% of all parasympathetic nerve fibers. Postganglionic fibers innervate the atria in cardiac fat pads through ganglia, and subsequent neurotransmission is regulated by a nicotinic receptor. CAN affects the distal segment of the vagus nerve first, similar to the length-dependent aspect of diabetic peripheral neuropathy. A decrease in parasympathetic autonomic tone is largely evident in early CAN, as evaluated by cardiac autonomic reflex tests, and is accompanied by a reduction in heart rate variability (HRV). As a result, the sympathetic nervous system predominates, as seen by a greater resting heart rate. Autonomic nervous system imbalance emerges early in CAN pathogenesis and is related to increased cardiovascular risk prior to the development of definitive CAN. Insulin resistance, a hallmark of type 2 DM, prediabetes, and metabolic syndrome, exacerbates sympathetic predominance. Furthermore, ambulatory blood pressure monitoring reveals a lack of circadian rhythm in CAN patients with significant nocturnal hypertension. Untreated, parasympathetic vagal dysfunctions worsen, leading to the development of resting tachycardia caused by an unopposed sympathetic tone. Palpitations may be a common symptom of patients in this state, although they may also be entirely asymptomatic. As a result of advanced CAN, autonomic neuropathy develops in the (shorter) sympathetic plexus, with diminished sympathetic postural reflexes leading to postural hypotension. In end-stage CAN, the heart is completely denervated, and the resting increased heart rate is stable.

### 3.6. Alteration in Substrate Utilization

The heart is the most metabolically demanding organ; thus, it operates by consuming whichever energy substrate it has direct access to in order to maintain its contractile performance [50]. The healthy adult heart receives 60–80% of its energy through mitochondrial β-oxidation of fatty acids, with glucose, ketone bodies, lactate, and amino acids also being used to a lesser degree [50]. It should be stressed that glucose and ketone bodies are more efficient substrates compared to free fatty acids (FFAs) and that a shift towards FFA oxidation, as observed in patients with DM or insulin resistance, reduces the cardiac contractile function as a result of lipotoxicity [51].

However, the healthy adult heart is very flexible in terms of its regulation of overall energy metabolism, in which it constantly switches between glucose for oxidative metabolism in response to increases in circulating insulin postprandially and fatty acids in response to elevated plasma FFAs during prolonged fasting or starvation.

FAs can passively transfer across the cardiomyocytic plasma membrane to be utilized for cardiac ATP production, but their absorption is aided by both the FA translocase (CD36) and the FA binding protein (FABP) [52]. Carnitine palmitoyltransferase I (CPT1) converts FAs in the cytosol to long-chain acylcarnitine before they enter the mitochondria, where they are reversed to acyl-CoA by CPT2 and undergo β-oxidation [52]. Cytosolic FAs, on the other hand, can be esterified to produce triglycerides for storage in the myocardial triglyceride (TG) pool [52]. Despite the heart’s reliance on FAs for ATP, overall TG storage capacity is limited, with only around 3 mg of TGs stored per gram of myocardial tissue [50]. This could be due to the fact that excessive lipid accumulation can exert deleterious effects on the heart. 

HF is exacerbated by changes in cardiac energy metabolism. The metabolic alterations that occur in HF, on the other hand, are complicated and rely not only on the degree and type of HF but also on the presence of common comorbidities such as obesity and type 2 DM. The failing heart has an energy deficit, which is caused mostly by a decline in mitochondrial oxidative capability. This is offset in part by an increase in ATP synthesis from glycolysis. The proportional contribution of various fuels to mitochondrial ATP synthesis changes as well, with glucose and amino acid oxidation decreasing and ketone oxidation increasing. Depending on the kind of HF, the heart’s fatty acid oxidation augments or decreases. For example, concerning HFpEF caused by diabetes or obesity, myocardial fatty acid oxidation increases [53,54], but in heart failure caused by hypertension or ischemia, it decreases. These energy-metabolic alterations, when combined, cause the failing heart to become less efficient.

## 4. The Role of Diabetes Mellitus in Heart Failure Pathophysiology

HF among individuals with DM is induced by the dysregulated management of glucose and insulin. The ultimate observation is the impairment of systolic and diastolic cardiac function by the reduction in myocardial contractility and compliance.

### 4.1. Myocardial Inflammation in Diabetes Mellitus

The role of inflammation in DM-induced cardiac dysfunction has already been established. In a recently reported experimental study, investigators detected an increase in MyD88-TLR2-TLR4 expression of primary cardiomyocytes, which was paired with an overexpression of inflammatory genes such as *IL-1b*, *IL-6*, and *Tnfa* [55]. These findings indicate the role of nuclear factor-kappaB (NF-κB), a major mediator of inflammation, in diabetic cardiomyopathy. Moreover, the authors proceeded to conduct an in vivo experiment using db/db mice, thereby confirming the in vitro findings. Advanced glycation end-products (AGEs) are also critical to NF-κB activation and myocardial inflammation in DM [56]. These types of molecules form when proteins or fats in the body become glycated. Their formation or binding to the receptor for AGEs (RAGE) expressed on the myocardial cell surface can induce inflammatory signaling. Moreover, RAGE signaling in endothelial cells and monocytes can also indirectly and adversely affect the myocardium [57]. The importance of AGEs in HF pathophysiology was recently demonstrated by the reported outcomes of the Rotterdam study [58]. The investigators detected an association between AGEs, whose presence is assessed by skin autofluorescence, and the prevalence of HF, which was more potent among individuals with DM. Interestingly, increasing AGEs were associated with lower LVEF in subjects without an HF diagnosis.

Following NF-κB activation, deleterious inflammatory processes ensue in the myocardium of patients with DM, including cardiomyocyte hypertrophy, fibrosis, and apoptosis, as well as the impairment of myocardial energetics, calcium management, and contractility [59]. Several human studies have suggested that an augmented systemic inflammatory burden could be predictive of incident HF in individuals with DM. In the ADVANE trial of 3098 participants with type 2 DM, one standard deviation increase in interleukin-6 (IL-6) (hazard ratio: 1.48, 95% confidence interval: 1.27 to 1.72) or high sensitivity CRP (hazard ratio: 1.32, 95% confidence interval: 1.12 to 1.55) was associated with a higher risk of HF incidence or progression [60].

### 4.2. Myocardial Fibrosis in Diabetes Mellitus

Numerous factors, such as oxidative stress, pro-inflammatory states, growth factor secretion, neurohumoral activation, the deposition of AGEs, and the activation of the RAAS, may raise the likelihood of diffuse myocardial fibrosis among people with DM [61]. The activation of myofibroblasts, which generate fibrous tissue, by these effectors may alter the balance of fibrosis deposition throughout breakdown [40]. Studies, both preclinical and clinical, have shown that DM induces a pro-fibrotic state. Four-week-old male Sprague–Dawley rats with streptozocin-induced DM exhibited a higher collagen type I and III accumulation compared to the control group [62]. Critically, a recently published systematic review and meta-analysis of 32 studies by Salvador et al. highlighted a relationship between DM and a higher degree of myocardial fibrosis assessed by either the histological collagen volume fraction (mean difference 5.80; 95% CI: 2.00–9.59) or extracellular volume fraction (mean difference: 2.09; 95% CI: 0.92–3.27) [63]. The findings were more pronounced in individuals with worse glycemic control [63]. It should also be stated that increasing myocardial fibrosis assessed by cardiac magnetic resonance imaging could signify an augmented risk of major adverse cardiac, cerebrovascular, and heart failure events in individuals with DM [64,65].

### 4.3. Cardiac Autonomic Neuropathy in Diabetes Mellitus

The presentation of dysglycemia and insulin resistance in individuals with DM are plausible mediators of excessive sympathetic activity, which leads to myocardial hypertrophy, fibrosis, and dysfunction, thereby easing progression to HF [66]. Hyperglycemia is assumed to be the chief factor, as it triggers a series of complicated processes and pathways that cause oxidative stress and toxic glycosylation products, ultimately leading to neuronal malfunction and death [67]. Hyperglycemia causes an increase in the mitochondrial synthesis of free radicals, which causes oxidative damage to the microvasculature that supplies these peripheral nerves [68]. Inflammation may be an additional driver of CAN, as indicated by increased levels of inflammatory molecules in those with CAN [69]. Moreover, increased IL-1 levels could predict progressive changes in the resting heart rate of individuals with type 2 DM, which is suggestive of inflammation’s role in the development of CAN [70]. However, the exact pathophysiology of CAN is unknown, since the processes involved have only been examined in somatic models and extended to the autonomic nervous system.

The criteria for the diagnosis of CAN among DM patients has not been established, and usually requires one to two abnormal functional tests (excitation/inhibition ratio, the Valsalva maneuver, 30:15 ratio, blood pressure postural decrease, and HRV indices). Its prevalence ranges from 17% to 73% in individuals with DM [69], and may also be increased in those with prediabetes compared to normoglycemic subjects [71]. The severity of CAN is dependent on the duration of DM, the degree of glycemic control, the presence of comorbidities, and specific genetic polymorphisms [72,73,74]. Moreover, the role of CAN in the development of HF was recently documented in the ACCORD study [75]. Analyzing 7160 participants without HF at baseline over a 4.9-year follow-up, the investigators highlighted that lower HRV measured using standard deviation of all normal-to-normal intervals (SDNN) led to a greater incident HF risk, even after adjustment for confounders (adjusted hazard ratio for lowest vs. highest SDNN quartile—1.70; 95% confidence interval—1.14 to 2.54). Moreover, individuals with CAN, defined as the lowest SDNN quartile with the highest QT interval index and heart rate quartiles, had an augmented risk of developing HF (adjusted hazard ratio—2.65; 95% confidence interval—1.57 to 4.48).

### 4.4. Cardiac Lipotoxicity in Diabetes Mellitus

Due to a ‘lipid spillover’ from overloaded adipose tissue, circulating free FAs (FFAs) and triglyceride (TG)-rich very low-density lipoproteins (VLDL) are elevated in DM sufferers, thus enhancing their availability for absorption by the heart. Elevated plasma FFA concentrations result in considerably increased myocardial FFA absorption and consequent intramyocardial lipid deposition [76], indicating that serum FFAs are important regulators of myocardial lipid accumulation. Furthermore, in type 2 DM, lipoprotein lipase (LPL) expression on cardiomyocytes is increased, thus improving VLDL hydrolysis in the coronary circulation and thereby increasing lipid availability [77]. As a result, lipid absorption by cardiomyocytes increases in DM-affected individuals, possibly stimulating an increase in FA oxidation. This increase in FA oxidation is likely further provoked by decreased glucose uptake induced by insulin resistance, as well as the suppression of glucose oxidation by lipid catabolism intermediates such as acetyl-CoA and citrate [78].

Despite this increase in FA oxidation, research shows that it is frequently insufficient to avoid myocardial TG buildup in patients with HFpEF. HFpEF patients showed 2.3-fold greater myocardial lipid content than the control participants in research utilizing CMR to measure cardiac lipids, and this content was independently linked with diastolic strain rate [79]. Similar research has shown that HFpEF patients have considerably more intramyocardial fat than non-HF and HFrEF patients, which corresponds to both the E/e’ ratio and the left atrial volume index, while women with subclinical HFpEF had more intramyocardial fat than reference controls [80,81]. A very low-calorie diet causes an initial increase in myocardial TG deposition, which is related to immediate diastolic functional impairment as measured by a lower E/A ratio in type 2 DM patients [76]. This group of patients also had a 2.1-fold higher myocardial TG content than the normoglycemic controls and a worse early diastolic filling rate [82]. These findings suggest that intramyocardial fat accumulation is linked to decreased diastolic performance among people with diabetes.

## 5. A Novel Era in Heart Failure Pharmacotherapy: SGLT2 Inhibitors

As the interaction of DM with HF is of particular interest, the effect of novel antidiabetic agents has been examined in HF populations, and the foremost of such agents is SGLT2i. Below, we elaborate on their pleiotropic mechanisms of action that could be of importance in reverting HF pathophysiology, as shown in preclinical studies. Moreover, we provide the available information stemming from clinical trials and cohort studies that demonstrate the effect of SGLT2i on the prevention of adverse HF outcomes.

### 5.1. SGLT2i as Hypoglycemic Agents

The SGLT2 protein consists of a glucose ring and a proximal and distal benzene ring connected by a methylene bridge. As they are responsible for the reabsorption of the majority of filtered glucose by the glomeruli, SGLT1 and SGLT2, which are found on the proximal convoluted tubule of nephrons, are essential mediators of glucose homeostasis. The large capacity, low-affinity SGLT2 absorbs 90% of the glucose, with a sodium turnover ratio of 1:1 [83,84]. As a result, facilitative glucose transporters (GLUTs) situated in the epithelial lining of proximal tubules unleash glucose into the bloodstream [83]. The impact of SGLT inhibition on diabetes was first observed in 1987 with the use of phlorizin, a non-specific SLGT1 and SGLT2 inhibitor [85]. Its treatment resulted in reduced hyperglycemia and increased glycosuria in an animal model of type 2 DM, along with the maintenance of normal insulin sensitivity. However, specific SGLT2i types have also been produced. Dapagliflozin was the first high-potency SLGT2i to be discovered [86], followed by canagliflozin [87], empagliflozin [88], and ertugliflozin [89]. Other compounds of this family, such as ipragliflozin, tofogliflozin, and luseogliflozin, have also been investigated, but less thoroughly. In terms of hypoglycemic impact, all licensed medications have shown efficacy in terms of decreasing glucose levels and glycated hemoglobin (HbA1c) in randomized clinical studies.

### 5.2. Pleiotropic Mechanisms of SGLT2i

Several mechanisms are thought to be involved in the pleiotropic effects conferred by SGLT2 inhibition to HF (Table 1). To begin with, among the earliest hypothesized processes to be identified were increased glycosuria and natriuresis, which led to osmotic diuresis and volume control. However, based on current SGLT2i HF studies, it is extremely unlikely that this theory captures the major driver of decreased HF morbidity. Although there may be a brief rise in diuresis [90], there is no effect on natriuretic peptides during the early phase [91]. This suggests that a greater proportion of patients with a moderate or significant decrease in natriuretic peptides during long-term follow-up may be undergoing a reversal of the unfavorable cardiac remodeling process [92]. As we recently showed through a systematic review and meta-analysis, the use of SGLT2i led to significant improvement of systolic and diastolic function-imaging indices [93], which is indicative of the amelioration of cardiac remodeling that was effected through this treatment.

**Figure 1:**
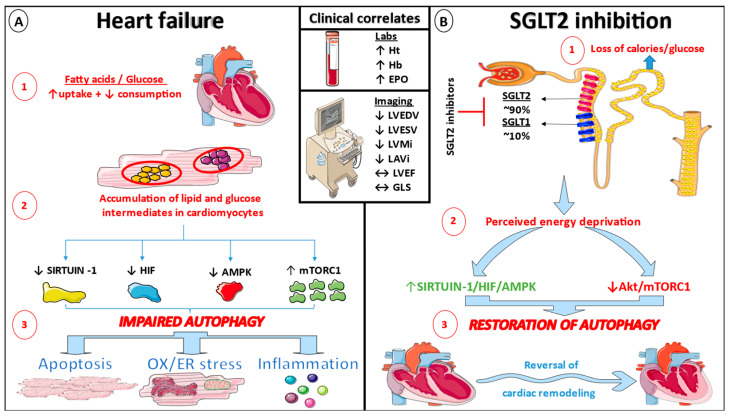
Impaired autophagy in heart failure and the potential role of SGLT2 inhibition. (**A**) Heart failure is a nutrient excess disease that promotes the buildup of lipid and glucose intermediates in cardiomyocytes, resulting in defective autophagy, apoptosis, oxidative (OX) and endoplasmic reticulum (ER) stress, and inflammation. (**B**) SGLT2 inhibitors trigger a state of apparent nutritional shortage by altering the equilibrium between the Sirtuin-1/Hypoxia-inducible factor (HIF)/AMPK pathway and the Akt/mammalian target of rapamycin complex 1 (mTORC1) pathway. As a result, restoring autophagy benefits the reversal of unfavorable cardiac remodeling. ↑ indicates an increase, ↓ indicates a decrease.

At this point, it should be noted that SGLT2 expression is lacking in the human heart, but SGLT1 is plentiful, particularly in pathologic conditions [109]. Nonetheless, the cardioprotective effects seen with even highly selective SGLT2i (empagliflozin) may suggest that these benefits are primarily regulated by indirect, systemic actions [110]. The emergence of hemoglobin and hematocrit as the most crucial predictors of the improvements reported with SGLT2-is, according to statistical mediation studies of major clinical trials, has led to a reassessment of the probable mechanistic relationship [111,112]. The impact of SGLT2 inhibition on erythropoiesis was investigated further in a sub-study of the EMPA-HEART CardioLink-6 randomized controlled trial (RCT), which revealed an increase in erythropoietin, hemoglobin, and hematocrit six months after randomization [113]. Since an increase in hematocrit and hemoglobin alone did not result in cardiovascular benefits when erythropoietin-like agents were used, this effect could be driven by hypoxia-inducible factors (HIFs) [114], potentially as an additional result of the stimulation of nutrient deprivation signaling pathways [115]. It has long been recognized that DM and HF are conditions characterized by a nutrient surplus, which leads to the inhibition of the nutrient deprivation sirtuin-1 (SIRT1)/HIF/5′ adenosine monophosphate-activated protein kinase (AMPK) pathway and the stimulation of the Akt/mammalian target of rapamycin complex 1 (mTORC1) pathway, resulting in increased endoplasmic reticulum stress, oxidative stress, inflammation, and apoptosis (Figure 1). According to a novel concept, SGLT2 inhibition may cause caloric loss in the urine and a reduced tissue distribution of glucose, resulting in a global oxygen and nutrient deficiency and, as a result, affecting the balance between the pathways listed above [29]. Indeed, SGLT2 expression appears to be negatively associated with SIRT1 in the proximal renal tubular area [30]. In a recent in vitro study of angiotensin II-stimulated cardiomyocytes, dapagliflozin activated SIRT1 and attenuated HF development by inhibiting the transformation of fibroblasts into myofibroblasts as well as fibroblast migration [94]. The study of Yu et al. also suggested that SGLT2i could alleviate myocardial injury by restoring autophagy, but other recent studies provided conflicting results [116,117]. Therefore, the existing evidence is not straightforward, and it may be the mediation of the selective autophagic flux that is critical. Lastly, the latest report of the EMPEROR program analyzing circulating proteomics supports the validity of the widespread effect of empagliflozin on the amelioration of autophagic flux at the heart, kidneys, and endothelium [118]. 

SGLT2-Is may be able to improve oxidative stress and inflammation [119,120]. Antioxidant and anti-inflammatory effects of SGLT2 inhibition have been demonstrated in recent experimental studies at the level of the heart [96,97], implicating key inflammatory mediators such as the NLRP3 inflammasome and NF-κB. According to our systematic review and meta-analysis, SGLT2i can significantly decrease the levels of circulating inflammatory markers, thereby confirming this potential anti-inflammatory action [121]. Furthermore, in a nine-week investigation of pigs with HF and retained LVEF, Zhang et al. found that daily dapagliflozin treatment resulted in decreased expression of inflammatory markers in the aortic tissue [98]. After discovering high expression of the SGLT1 isoform in human cardiomyocytes (SGLT2 was undetectable), Kondo et al. performed SGLT inhibition with canagliflozin [99]. The researchers discovered antioxidant effects such as decreased nicotinamide adenine dinucleotide phosphate oxidase activity, increased tetrahydrobiopterin bioavailability, and enhanced NO synthase coupling via a mechanism involving SGLT1/AMPK/Rac1. Additional anti-inflammatory and anti-apoptotic effects were detected. However, there is a scarcity of evidence in the clinical context. Although other studies found a substantial reduction in oxidative stress (8-iso-prostaglandin F2a and 8-hydroxy-20-deoxyguanosine) and inflammatory markers (CRP, IL-6, and tumor necrosis factor-alpha (TNF-α)), their designs were not capable of demonstrating the correctness of those findings [122]. In a study of patients who underwent coronary artery bypass grafting, those who received SGLT2i had significantly reduced IL-1, IL-6, and TNF-α levels at the 5-year follow-up [123]. It should be noted that a few studies [124,125,126,127] have revealed non-significant changes in the aforementioned parameters. However, in the EMPA-TROPISM study, 6-month therapy with empagliflozin resulted in a substantial decrease in inflammatory biomarkers in patients without diabetes, with HF, and a lower LVEF [128]. A recent systematic review and meta-analysis of RCTs has also suggested a significant anti-inflammatory effect of SGLT2 inhibition [129]. Additionally, the proteomic analysis on empagliflozin trials has also shown a differential expression of proteins associated with inflammation and oxidative stress at the level of the heart [118].

Fibrosis may also be reduced after SGLT2 inhibition, as indicated by decreased collagen deposition in cardiac cells after therapy with dapagliflozin or empagliflozin [100,101]. Furthermore, treatment with sotagliflozin, a dual SGLT1/2 inhibitor, resulted in reduced histological fibrosis in a mouse model of transverse aortic constriction-induced pressure overload [102]. In the same study, dapagliflozin reduced left ventricular fibrosis in a rat model of mitral regurgitation-induced myocardial failure, which was caused by decreased apoptosis and endoplasmic reticulum stress [130]. Tian et al. observed a reduction in cardiac fibrosis after treatment of dapagliflozin in a mouse model of diabetic cardiomyopathy, as evidenced by decreased collagen deposition and levels of fibrosis biomarkers [103]. The reduced endothelial-to-mesenchymal transition and fibroblast activation were identified as the orchestrators of this positive impact via the AMPK/TGF-β/Smad signaling pathway [103]. This signaling pathway was also linked to dapagliflozin’s anti-fibrotic actions in a rat model of angiotensin-II-induced heart failure [104]. Empagliflozin therapy resulted in the AMPK-mediated alleviation of myocardial fibrosis in a post-myocardial infarction animal model [105]. In addition, in a multimodality investigation of pigs with proximal left anterior descending artery occlusion-induced HF, empagliflozin treatment was linked with improved diastolic function based on decreased fibrosis and endothelial dysfunction [106]. There is a paucity of clinical evidence on the effect of SGLT2 inhibition on fibrosis. Daily empagliflozin administration for six months showed no effect on fibrosis as measured by cardiac magnetic resonance among type 2 DM patients [131]. Patients with type 2 DM and CAD experienced a decrease in extracellular volume in the randomized controlled experiment EMPA-HEART CardioLink-6 [132]. Finally, compared to the control group, participants in the EMPA-TROPISM study showed a substantial reduction in interstitial myocardium fibrosis following chronic therapy with empagliflozin [128]. Proteins associated with fibrosis were among those with altered cardiac expression in a proteomic signature analysis reported by the EMPEROR program, which further supports the anti-fibrotic hypothesis of SGLT2i [118].

Other mechanisms involved in the deleterious effect of DM on the heart may be influenced by SGLT2 inhibition but have been studied to a lesser extent. SGLT2i may attenuate JunD/PPAR-γ-mediated cardiac lipotoxicity [107]. Canagliflozin also ameliorated cardiomyocyte lipotoxicity through the mTOR/HIF-1α pathway in an experimental study conducted by Sun et al. [108]. Another explanatory concept is the decrease in epicardial fat caused by leptin downregulation [128,133]. Epicardial adipose tissue growth is linked to poor left ventricular relaxation and diastolic filling, either directly through constriction or indirectly through the regulation of pro-inflammatory molecule release, resulting in inflammation, microcirculatory dysfunction, and fibrosis [134]. SGLT2 expression has been established in human epicardial adipose tissue, and the majority of the data on individuals with type 2 DM show a decrease in epicardial adipose tissue mass after SGLT2 inhibition [134,135]. The newly published randomized, placebo-controlled EMPA-TROPISM study found that empagliflozin decreased epicardial fat content in individuals without T2DM, HF, and a lower LVEF [128]. At the same time, another molecular theory has been questioned, namely, the impact of SGLT2 inhibitors on the cardiac sodium–hydrogen exchanger-1 as a means of minimizing myocardial damage and incident HF [136,137,138]. Enhanced ATP generation owing to ketone body overproduction through beta-hydroxybutirate metabolism was an intriguing idea in this regard, since ketones are considered to be a more efficient substrate. However, this was not confirmed in a recent experiment [139]. Moving on to the effects of SGLT2i on CAN, in a study of patients with type 2 DM and vasovagal syncope recurrence, SLGT2i use was associated with a significantly lower low frequency/high frequency ratio and norepinephrine serum levels and a higher Heart to Mediastinum ratio in 123I-metaiodobenzylguanidine (123I-mIBG) myocardial scintigraphy at the conclusion of the study [140]. These SGLT2i-mediated effects might indicate more balanced autonomic system activity and an induced improvement in CAN. Dapagliflozin also ameliorated heart rate variability and heart rate turbulence parameters in patients with type 2 DM and CAN [141]. These findings contradict a recent meta-analysis that documented a trivial effect of SGLT2 inhibition on indices of CAN [142]. It should be noted that the improvement in CAN with SGLT2i may be more pronounced in individuals with HF, as suggested by the study of Hamaoka et al. [143]. Finally (but still of great importance), an improvement in renal function observed with SGLT2i may be responsible for some of the cardiac benefits [144].

### 5.3. SGLT2 Inhibitors and Heart Failure Outcomes

**Figure 2:**
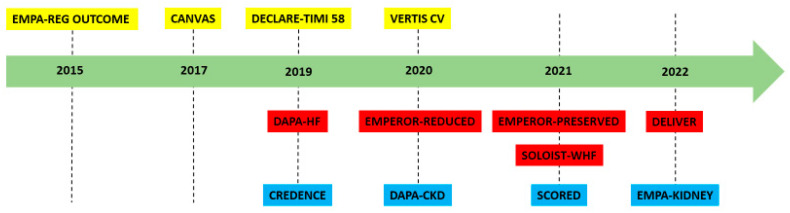
Timeline of landmark SGLT2 inhibitor randomized clinical trials. Yellow indicates cardiovascular outcome trials, red indicates trials among heart failure populations, and blue indicates trials among chronic kidney disease populations.

The programs of SGLT2i clinical trials have met with overwhelming success concerning HF outcomes, either in trials of solely HF populations or in studies of patients with DM or kidney diseases (Figure 2). Following the initial positive findings in cardiovascular outcome trials, research into HF patients was conducted to explore the working hypothesis of cardiac protection further. The DAPA-HF study found a decreased risk of worsening HF or cardiovascular mortality in patients with HF and a lowered LVEF, which were independent of DM status [145]. Notably, there was no difference in outcomes based on HF etiology (ischemic vs. non-ischemic) [146], the simultaneous use of an angiotensin receptor neprilysin inhibitor (ARNI) [147], or baseline health condition [148]. Simultaneously, patients with longer HF duration [149] and LVEF of 35% saw more substantial benefits [150]. Following therapy with dapagliflozin, the participants’ quality of life improved significantly [148]. The subsequent EMPEROR-REDUCED study investigating a comparable patient population found decreased rates of cardiovascular mortality and HF hospitalization in the empagliflozin arm compared to the placebo, regardless of DM [151]. In terms of the co-administration of agents proven to be effective for the management of HF with reduced LVEF, the use of mineralocorticoid receptor antagonists (MRAs) did not change the initial findings, whereas treatment with empagliflozin resulted in lower MRA discontinuation rates and higher MRA initiation rates [111]. Regarding angiotensin receptor neprilysin inhibitors (ARNIs), their usage was related to an incremental decline in endpoint occurrence [152]. Furthermore, the outcomes were constant regardless of chronic kidney disease status or baseline HbA1c [153,154].

SGLT2 inhibition has also proven to be effective in terms of the difficult-to-treat HF population with maintained LVEF, a condition with no previously authorized therapeutic options other than symptomatic. In the SOLOIST-WHF study, starting sotagliflozin after an episode of worsening HF resulted in decreased risks of cardiovascular death, HF hospitalizations, or urgent visits. Despite the fact that the study was prematurely discontinued and did not specifically investigate the effect of sotagliflozin on HF with preserved LVEF, a 52% relative risk reduction in the primary endpoint was observed in patients with HF with LVEF 50% on sotagliflozin [155]. The EMPEROR-PRESERVED trial findings corroborated the efficacy of SGLT2 inhibition towards HF with LVEF >40%, indicating a 29% reduction in HF hospitalizations associated with an improvement in patients’ quality of life, regardless of the presence of DM [156]. Despite the fact that patients with modestly decreased LVEF were also included in the study, the results were fairly constant across the LVEF range, with the highest advantages shown in those with LVEF 50% or lower. In the DELIVER trial of dapagliflozin, individuals with HFmrEF or HFpEF receiving the intervention experienced lower endpoint rates compared to those receiving a placebo [157]. Dapagliflozin’s effect was evident as early as 2 weeks after initiation [158], was more pronounced in subjects with a worse baseline symptom burden [159], and was consistent irrespective of age, body mass index, frailty, baseline glycemic status, or MRA/ARNI use [160,161,162,163,164]. The expected addition of SGLT2i in the societal guidelines of HFpEF/HFmrEF management may result in a significant alleviation of HF burden, with a recent study indicating 250,000 fewer hospitalizations for worsening HF in this group of patients [165].

## 6. Conclusions

Diabetes mellitus represents a major risk factor for the development of heart failure since the two afflictions’ pathophysiological characteristics are closely related, as proven by multiple preclinical and clinical studies. The emergence of a diabetic pharmacotherapy, particularly the use of sodium-glucose cotransporter-2 inhibitors, as an effective approach in the management of heart failure across the spectrum of left ventricular ejection fraction highlights the need for their implementation in real-world clinical practice to reduce the burden of both diseases.

## Figures and Tables

**Table 1:** Preclinical evidence of the pleiotropic mechanisms of action of SGLT2 inhibitors (SGLT2i).

	Type	Experimental Model	Disease Type	SGLT2i	Finding	Target Mechanism
Ren et al. [94]	In vitro	Cardiomyocytes	HF	DAPA	↑ SIRT1 ↓ fibroblast transformation to myofibroblast ↓ fibroblast migration	Autophagy Fibrosis
Yu et al. [95]	In vitro + In vivo	Primary cardiomyocytes Male C57/BL6 mice	IRI	DAPA	↓ IL-1β ↓ NLRP3 inflammasome activation ↓ Atg-5, Beclin-1, LC3B-II and P62 ↑ NLRP3 phagocytosis by autophagosomes	Inflammation Autophagy
Quaqliariello et al. [96]	In vivo	C57Bl/6 mice	Cardiotoxicity	EMPA	↓ intracellular ROS, MDA, lipid peroxidation↓ IL-6, IL-8, NF-κB, NLRP3 ↓ pro-collagen 1α1, MMP-9 ↓ apoptotic nuclei, caspase-3	Oxidative stress Inflammation Fibrosis Apoptosis
Sukhanov et al. [97]	In vitro	Aortic SMC	Inflammation	EMPA	↓ NLRP3, IL-1β, IL-18, Caspase-1 ↓ Superoxide, hydrogen peroxide	Inflammation Oxidative stress
Zhang et al. [98]	In vivo	Female landrace pigs	HF	DAPA	↓ Collagen-1 and -3, TGF-β	Fibrosis
Kondo et al. [99]	Ex vivo + In vitro	Atrial tissue H9C2 and primary human cardiomyocytes	High glucose	CANA	↓ NADPH ↑ BH4, NOS coupling ↓ NF-κB, TNF-α, and apoptosis pathways	Oxidative stress Inflammation Apoptosis
Cappetta et al. [100]	In vivo + In vitro	Dahl salt-sensitive rats Ventricular cardiomyocytes	Hypertension	DAPA	↓ VCAM-1, E-Selectin ↓ NF-κB, IL-6, MCP-1 ↓ Collagen-1, TGF-β, MMP-2	Endothelial dysfunction Inflammation Fibrosis
Madonna et al. [101]	In vivo	Male C57BL/6 mice	DM	EMPA	↓ collagen content ↓ p38	Fibrosis Inflammation
Young et al. [102]	In vivo	Male C57BL/6J mice	HF	SOTA	↓ histological fibrosis	Fibrosis
Tian et al. [103]	In vivo + In vitro	Male Sprague Dawley rats HUVECs	DM	DAPA	↓ collagen deposition ↓ TGF-β expression ↓ EndMT ↓ fibroblast activation ↓ ROS and NADPH Oxidase 4	Fibrosis Oxidative stress
Zhang et al. [104]	In vivo	Sprague-Dawley rats	HF	DAPA	↓ Collagen-1 and -3, TGF-β	Fibrosis
Liu et al. [105]	In vivo	C57BL/6 mice	HF	EMPA	↓ histological fibrosis ↓ caspase-3, Bcl2	Fibrosis Apoptosis
Santos-Gallego et al. [106]	In vivo	Yorkshire pigs	HF	EMPA	↓ histological and imaging fibrosis ↑ NO bioavailability, cGMP, PKG	Fibrosis Endothelial dysfunction
Marfella et al. [107]	In vitro	Ventricular cardiomyocytes	DM	Any	↑ JunD/PPAR-γ and ceramide ↓ IRS1 and IRS2	Lipotoxicity
Sun et al. [108]	In vivo + In vitro	C57BL/6J mice HL-1 cells	DM	CANA	↓ IL-6 and TNF-α ↓ ROS ↓ mTOR or HIF-1α signaling	Inflammation Oxidative stress Lipotoxicity

HF: heart failure, DAPA: dapagliflozin, IRI: ischemia/reperfusion injury, IL: interleukin, EMPA: empagliflozin, ROS: reactive oxygen species, MDA: malondialdehyde, NF-κB: nuclear factor-kappaB, MMP: matrix metalloproteinase, SMC: smooth muscle cell, TGF: transforming growth factor, BH4: tetrahydrobiopterin, CANA: canagliflozin, NADPH: nicotinamide adenine dinucleotide phosphate, TNF: tumor necrosis factor, VCAM: vascular cell adhesion molecule, MCP: monocyte chemoattractant protein, DM: diabetes mellitus, SOTA: sotagliflozin, EndMT: endothelial-to-mesenchymal transition, NO: nitric oxide, PKG: protein kinase G, PPAR: peroxisome proliferator-activated receptor, IRS: insulin receptor substrate, mTOR: mammalian target of rapamycin, and HIF: hypoxia-inducible factor. ↑ indicates an increase, ↓ indicates a decrease.

## Data Availability

Not applicable.

## References

[1] Saeedi P., Petersohn I., Salpea P., Malanda B., Karuranga S., Unwin N., Colagiuri S., Guariguata L., Motala A.A., Ogurtsova K. (2019). Global and regional diabetes prevalence estimates for 2019 and projections for 2030 and 2045: Results from the International Diabetes Federation Diabetes Atlas, 9(th) edition. Diabetes Res. Clin. Pract..

[2] Ling W., Huang Y., Huang Y.M., Fan R.R., Sui Y., Zhao H.L. (2020). Global trend of diabetes mortality attributed to vascular complications, 2000-2016. Cardiovasc. Diabetol..

[3] Einarson T.R., Acs A., Ludwig C., Panton U.H. (2018). Prevalence of cardiovascular disease in type 2 diabetes: A systematic literature review of scientific evidence from across the world in 2007–2017. Cardiovasc. Diabetol..

[4] McAllister D.A., Read S.H., Kerssens J., Livingstone S., McGurnaghan S., Jhund P., Petrie J., Sattar N., Fischbacher C., Kristensen S.L. (2018). Incidence of Hospitalization for Heart Failure and Case-Fatality Among 3.25 Million People With and Without Diabetes Mellitus. Circulation.

[5] Ciardullo S., Cannistraci R., Mazzetti S., Mortara A., Perseghin G. (2022). Twenty-year trends in heart failure among U.S. adults, 1999–2018: The growing impact of obesity and diabetes. Int. J. Cardiol..

[6] Leung A.A., Eurich D.T., Lamb D.A., Majumdar S.R., Johnson J.A., Blackburn D.F., McAlister F.A. (2009). Risk of heart failure in patients with recent-onset type 2 diabetes: Population-based cohort study. J. Card Fail.

[7] Lecoeur E., Domenge O., Fayol A., Jannot A.S., Hulot J.S. (2022). Epidemiology of heart failure in young adults: A French nationwide cohort study. Eur. Heart J..

[8] Wamil M., Coleman R.L., Adler A.I., McMurray J.J.V., Holman R.R. (2021). Increased Risk of Incident Heart Failure and Death Is Associated With Insulin Resistance in People With Newly Diagnosed Type 2 Diabetes: UKPDS 89. Diabetes Care.

[9] He J., Ogden L.G., Bazzano L.A., Vupputuri S., Loria C., Whelton P.K. (2001). Risk factors for congestive heart failure in US men and women: NHANES I epidemiologic follow-up study. Arch. Intern. Med..

[10] Thrainsdottir I.S., Aspelund T., Thorgeirsson G., Gudnason V., Hardarson T., Malmberg K., Sigurdsson G., Ryden L. (2005). The association between glucose abnormalities and heart failure in the population-based Reykjavik study. Diabetes Care.

[11] Kannel W.B., Hjortland M., Castelli W.P. (1974). Role of diabetes in congestive heart failure: The Framingham study. Am. J. Cardiol..

[12] Ohkuma T., Komorita Y., Peters S.A.E., Woodward M. (2019). Diabetes as a risk factor for heart failure in women and men: A systematic review and meta-analysis of 47 cohorts including 12 million individuals. Diabetologia.

[13] Chioncel O., Lainscak M., Seferovic P.M., Anker S.D., Crespo-Leiro M.G., Harjola V.P., Parissis J., Laroche C., Piepoli M.F., Fonseca C. (2017). Epidemiology and one-year outcomes in patients with chronic heart failure and preserved, mid-range and reduced ejection fraction: An analysis of the ESC Heart Failure Long-Term Registry. Eur. J. Heart Fail..

[14] Boonman-de Winter L.J., Rutten F.H., Cramer M.J., Landman M.J., Liem A.H., Rutten G.E., Hoes A.W. (2012). High prevalence of previously unknown heart failure and left ventricular dysfunction in patients with type 2 diabetes. Diabetologia.

[15] Sinha A., Ning H., Cameron N., Bancks M., Carnethon M.R., Allen N.B., Wilkins J.T., Lloyd-Jones D.M., Khan S.S. (2022). Atherosclerotic Cardiovascular Disease or Heart Failure: First Cardiovascular Event in Adults With Prediabetes and Diabetes. J. Card. Fail..

[16] Birkeland K.I., Bodegard J., Eriksson J.W., Norhammar A., Haller H., Linssen G.C.M., Banerjee A., Thuresson M., Okami S., Garal-Pantaler E. (2020). Heart failure and chronic kidney disease manifestation and mortality risk associations in type 2 diabetes: A large multinational cohort study. Diabetes Obes. Metab..

[17] Shah A.D., Langenberg C., Rapsomaniki E., Denaxas S., Pujades-Rodriguez M., Gale C.P., Deanfield J., Smeeth L., Timmis A., Hemingway H. (2015). Type 2 diabetes and incidence of cardiovascular diseases: A cohort study in 1.9 million people. Lancet Diabetes Endocrinol..

[18] Cannistraci R., Mazzetti S., Mortara A., Perseghin G., Ciardullo S. (2020). Risk stratification tools for heart failure in the diabetes clinic. Nutr. Metab. Cardiovasc. Dis..

[19] Niu X.N., Wen H., Sun N., Zhao R., Wang T., Li Y. (2022). Exploring risk factors of short-term readmission in heart failure patients: A cohort study. Front. Endocrinol..

[20] Pocock S.J., Wang D., Pfeffer M.A., Yusuf S., McMurray J.J., Swedberg K.B., Ostergren J., Michelson E.L., Pieper K.S., Granger C.B. (2006). Predictors of mortality and morbidity in patients with chronic heart failure. Eur. Heart J..

[21] Ciardullo S., Rea F., Cannistraci R., Muraca E., Perra S., Zerbini F., Mortara A., Perseghin G. (2022). NT-ProBNP and mortality across the spectrum of glucose tolerance in th e general US population. Cardiovasc. Diabetol..

[22] McDonagh T.A., Metra M., Adamo M., Gardner R.S., Baumbach A., Bohm M., Burri H., Butler J., Celutkiene J., Chioncel O. (2021). Corrigendum to: 2021 ESC Guidelines for the diagnosis and treatment of acute and chronic heart failure: Developed by the Task Force for the diagnosis and treatment of acute and chronic heart failure of the European Society of Cardiology (ESC) With the special contribution of the Heart Failure Association (HFA) of the ESC. Eur. Heart J..

[23] From A.M., Leibson C.L., Bursi F., Redfield M.M., Weston S.A., Jacobsen S.J., Rodeheffer R.J., Roger V.L. (2006). Diabetes in heart failure: Prevalence and impact on outcome in the population. Am. J. Med..

[24] Radhoe S.P., Veenis J.F., Linssen G.C.M., van der Lee C., Eurlings L.W.M., Kragten H., Al-Windy N.Y.Y., van der Spank A., Koudstaal S., Brunner-La Rocca H.P. (2022). Diabetes and treatment of chronic heart failure in a large real-world heart failure population. ESC Heart Fail..

[25] Dokainish H., Teo K., Zhu J., Roy A., AlHabib K.F., ElSayed A., Palileo-Villaneuva L., Lopez-Jaramillo P., Karaye K., Yusoff K. (2016). Heart Failure in Africa, Asia, the Middle East and South America: The INTER-CHF study. Int. J. Cardiol..

[26] Joseph P., Dokainish H., McCready T., Budaj A., Roy A., Ertl G., Gomez-Mesa J.E., Leong D., Ezekowitz J., Hage C. (2020). A multinational registry to study the characteristics and outcomes of heart failure patients: The global congestive heart failure (G-CHF) registry. Am. Heart J..

[27] Theofilis P., Sagris M., Antonopoulos A.S., Oikonomou E., Tsioufis C., Tousoulis D. (2021). Inflammatory Mediators of Platelet Activation: Focus on Atherosclerosis and COVID-19. Int. J. Mol. Sci..

[28] Sagris M., Theofilis P., Antonopoulos A.S., Tsioufis C., Oikonomou E., Antoniades C., Crea F., Kaski J.C., Tousoulis D. (2021). Inflammatory Mechanisms in COVID-19 and Atherosclerosis: Current Pharmaceutical Perspectives. Int. J. Mol. Sci..

[29] Oikonomou E., Leopoulou M., Theofilis P., Antonopoulos A.S., Siasos G., Latsios G., Mystakidi V.C., Antoniades C., Tousoulis D. (2020). A link between inflammation and thrombosis in atherosclerotic cardiovascular diseases: Clinical and therapeutic implications. Atherosclerosis.

[30] Adamo L., Rocha-Resende C., Prabhu S.D., Mann D.L. (2020). Reappraising the role of inflammation in heart failure. Nat. Rev. Cardiol..

[31] Suleiman M., Khatib R., Agmon Y., Mahamid R., Boulos M., Kapeliovich M., Levy Y., Beyar R., Markiewicz W., Hammerman H. (2006). Early inflammation and risk of long-term development of heart failure and mortality in survivors of acute myocardial infarction predictive role of C-reactive protein. J. Am. Coll. Cardiol..

[32] Nishimoto Y., Kato T., Morimoto T., Yaku H., Inuzuka Y., Tamaki Y., Yamamoto E., Yoshikawa Y., Kitai T., Taniguchi R. (2020). C-reactive protein at discharge and 1-year mortality in hospitalised patients with acute decompensated heart failure: An observational study. BMJ Open.

[33] Li X., Lin Y., Wang S., Zhou S., Ju J., Wang X., Chen Y., Xia M. (2020). Extracellular Superoxide Dismutase Is Associated With Left Ventricular Geometry and Heart Failure in Patients With Cardiovascular Disease. J. Am. Heart Assoc..

[34] Cao T.H., Jones D.J.L., Voors A.A., Quinn P.A., Sandhu J.K., Chan D.C.S., Parry H.M., Mohan M., Mordi I.R., Sama I.E. (2020). Plasma proteomic approach in patients with heart failure: Insights into pathogenesis of disease progression and potential novel treatment targets. Eur. J. Heart Fail..

[35] Lee C.F., Chavez J.D., Garcia-Menendez L., Choi Y., Roe N.D., Chiao Y.A., Edgar J.S., Goo Y.A., Goodlett D.R., Bruce J.E. (2016). Normalization of NAD+ Redox Balance as a Therapy for Heart Failure. Circulation.

[36] Theofilis P., Sagris M., Oikonomou E., Antonopoulos A.S., Siasos G., Tsioufis C., Tousoulis D. (2021). Inflammatory Mechanisms Contributing to Endothelial Dysfunction. Biomedicines.

[37] Zuchi C., Tritto I., Carluccio E., Mattei C., Cattadori G., Ambrosio G. (2020). Role of endothelial dysfunction in heart failure. Heart Fail. Rev..

[38] Dimitropoulos S., Mystakidi V.C., Oikonomou E., Siasos G., Tsigkou V., Athanasiou D., Gouliopoulos N., Bletsa E., Kalampogias A., Charalambous G. (2020). Association of Soluble Suppression of Tumorigenesis-2 (ST2) with Endothelial Function in Patients with Ischemic Heart Failure. Int. J. Mol. Sci..

[39] Fischer D., Rossa S., Landmesser U., Spiekermann S., Engberding N., Hornig B., Drexler H. (2005). Endothelial dysfunction in patients with chronic heart failure is independently associated with increased incidence of hospitalization, cardiac transplantation, or death. Eur. Heart J..

[40] Gonzalez A., Schelbert E.B., Diez J., Butler J. (2018). Myocardial Interstitial Fibrosis in Heart Failure: Biological and Translational Perspectives. J. Am. Coll. Cardiol..

[41] Webber M., Jackson S.P., Moon J.C., Captur G. (2020). Myocardial Fibrosis in Heart Failure: Anti-Fibrotic Therapies and the Role of Cardiovascular Magnetic Resonance in Drug Trials. Cardiol. Ther..

[42] Lim H., Zhu Y.Z. (2006). Role of transforming growth factor-beta in the progression of heart failure. Cell Mol. Life Sci..

[43] Armstrong A.C., Ambale-Venkatesh B., Turkbey E., Donekal S., Chamera E., Backlund J.Y., Cleary P., Lachin J., Bluemke D.A., Lima J.A. (2017). Association of Cardiovascular Risk Factors and Myocardial Fibrosis With Early Cardiac Dysfunction in Type 1 Diabetes: The Diabetes Control and Complications Trial/Epidemiology of Diabetes Interventions and Complications Study. Diabetes Care.

[44] Oikonomou E., Karlis D., Tsalamadris S., Siasos G., Chrysohoou C., Vogiatzi G., Dimitropoulos S., Charalambous G., Kouskouni E., Tousoulis D. (2019). Galectin-3 and Arterial Stiffness in Patients with Heart Failure: A Pilot Study. Curr. Vasc. Pharmacol..

[45] Krum H., Elsik M., Schneider H.G., Ptaszynska A., Black M., Carson P.E., Komajda M., Massie B.M., McKelvie R.S., McMurray J.J. (2011). Relation of peripheral collagen markers to death and hospitalization in patients with heart failure and preserved ejection fraction: Results of the I-PRESERVE collagen substudy. Circ. Heart Fail..

[46] Gulati A., Jabbour A., Ismail T.F., Guha K., Khwaja J., Raza S., Morarji K., Brown T.D., Ismail N.A., Dweck M.R. (2013). Association of fibrosis with mortality and sudden cardiac death in patients with nonischemic dilated cardiomyopathy. JAMA.

[47] Aoki T., Fukumoto Y., Sugimura K., Oikawa M., Satoh K., Nakano M., Nakayama M., Shimokawa H. (2011). Prognostic impact of myocardial interstitial fibrosis in non-ischemic heart failure—Comparison between preserved and reduced ejection fraction heart failure. Circ. J..

[48] Schelbert E.B., Piehler K.M., Zareba K.M., Moon J.C., Ugander M., Messroghli D.R., Valeti U.S., Chang C.C., Shroff S.G., Diez J. (2015). Myocardial Fibrosis Quantified by Extracellular Volume Is Associated With Subsequent Hospitalization for Heart Failure, Death, or Both Across the Spectrum of Ejection Fraction and Heart Failure Stage. J. Am. Heart Assoc..

[49] Duque A., Mediano M.F.F., De Lorenzo A., Rodrigues L.F. (2021). Cardiovascular autonomic neuropathy in diabetes: Pathophysiology, clinical assessment and implications. World J. Diabetes.

[50] Lopaschuk G.D., Ussher J.R., Folmes C.D., Jaswal J.S., Stanley W.C. (2010). Myocardial fatty acid metabolism in health and disease. Physiol. Rev..

[51] Glatz J.F.C., Nabben M., Young M.E., Schulze P.C., Taegtmeyer H., Luiken J. (2020). Re-balancing cellular energy substrate metabolism to mend the failing heart. Biochim. Biophys. Acta Mol. Basis Dis..

[52] Leggat J., Bidault G., Vidal-Puig A. (2021). Lipotoxicity: A driver of heart failure with preserved ejection fraction?. Clin. Sci..

[53] De Jong K.A., Lopaschuk G.D. (2017). Complex Energy Metabolic Changes in Heart Failure With Preserved Ejection Fraction and Heart Failure With Reduced Ejection Fraction. Can. J. Cardiol..

[54] Lopaschuk G.D., Karwi Q.G., Tian R., Wende A.R., Abel E.D. (2021). Cardiac Energy Metabolism in Heart Failure. Circ. Res..

[55] Luo W., Wu G., Chen X., Zhang Q., Zou C., Wang J., Liu J., Chattipakorn N., Wang Y., Liang G. (2022). Blockage of MyD88 in cardiomyocytes alleviates cardiac inflammation and cardiomyopathy in experimental diabetic mice. Biochem. Pharmacol..

[56] Cao W., Chen J., Chen Y., Chen S., Chen X., Huang H., Liu P. (2015). Advanced glycation end products induced immune maturation of dendritic cells controls heart failure through NF-kappaB signaling pathway. Arch. Biochem. Biophys..

[57] Bucciarelli L.G., Ananthakrishnan R., Hwang Y.C., Kaneko M., Song F., Sell D.R., Strauch C., Monnier V.M., Yan S.F., Schmidt A.M. (2008). RAGE and modulation of ischemic injury in the diabetic myocardium. Diabetes.

[58] Arshi B., Chen J., Ikram M.A., Zillikens M.C., Kavousi M. (2022). Advanced glycation end-products, cardiac function and heart failure in the general population: The Rotterdam Study. Diabetologia.

[59] Ramesh P., Yeo J.L., Brady E.M., McCann G.P. (2022). Role of inflammation in diabetic cardiomyopathy. Ther. Adv. Endocrinol. Metab..

[60] Ohkuma T., Jun M., Woodward M., Zoungas S., Cooper M.E., Grobbee D.E., Hamet P., Mancia G., Williams B., Welsh P. (2017). Cardiac Stress and Inflammatory Markers as Predictors of Heart Failure in Patients With Type 2 Diabetes: The ADVANCE Trial. Diabetes Care.

[61] Russo I., Frangogiannis N.G. (2016). Diabetes-associated cardiac fibrosis: Cellular effectors, molecular mechanisms and therapeutic opportunities. J. Mol. Cell Cardiol..

[62] Gao Z., Ti Y., Lu B., Song F.Q., Zhang L., Hu B.A., Xie J.Y., Zhang W., Han L., Zhong M. (2022). STAMP2 Attenuates Cardiac Dysfunction and Insulin Resistance in Diabetic Cardiomyopathy via NMRAL1-Mediated NF-kappaB Inhibition in Type 2 Diabetic Rats. Diabetes Metab. Syndr. Obes..

[63] Salvador D.B., Gamba M.R., Gonzalez-Jaramillo N., Gonzalez-Jaramillo V., Raguindin P.F.N., Minder B., Grani C., Wilhelm M., Stettler C., Doria A. (2022). Diabetes and Myocardial Fibrosis: A Systematic Review and Meta-Analysis. JACC Cardiovasc. Imaging.

[64] Yang Z., Xu R., Wang J.R., Xu H.Y., Fu H., Xie L.J., Yang M.X., Zhang L., Wen L.Y., Liu H. (2022). Association of myocardial fibrosis detected by late gadolinium-enhanced MRI with clinical outcomes in patients with diabetes: A systematic review and meta-analysis. BMJ Open.

[65] Wong T.C., Piehler K.M., Kang I.A., Kadakkal A., Kellman P., Schwartzman D.S., Mulukutla S.R., Simon M.A., Shroff S.G., Kuller L.H. (2014). Myocardial extracellular volume fraction quantified by cardiovascular magnetic resonance is increased in diabetes and associated with mortality and incident heart failure admission. Eur. Heart J..

[66] Thomas M.C. (2016). Type 2 Diabetes and Heart Failure: Challenges and Solutions. Curr. Cardiol. Rev..

[67] Vinik A.I., Erbas T., Casellini C.M. (2013). Diabetic cardiac autonomic neuropathy, inflammation and cardiovascular disease. J. Diabetes Investig..

[68] Pop-Busui R. (2010). Cardiac autonomic neuropathy in diabetes: A clinical perspective. Diabetes Care.

[69] Fisher V.L., Tahrani A.A. (2017). Cardiac autonomic neuropathy in patients with diabetes mellitus: Current perspectives. Diabetes Metab. Syndr. Obes..

[70] Hansen C.S., Vistisen D., Jorgensen M.E., Witte D.R., Brunner E.J., Tabak A.G., Kivimaki M., Roden M., Malik M., Herder C. (2017). Adiponectin, biomarkers of inflammation and changes in cardiac autonomic function: Whitehall II study. Cardiovasc. Diabetol..

[71] Ziegler D., Voss A., Rathmann W., Strom A., Perz S., Roden M., Peters A., Meisinger C., Group K.S. (2015). Increased prevalence of cardiac autonomic dysfunction at different degrees of glucose intolerance in the general population: The KORA S4 survey. Diabetologia.

[72] Spallone V. (2019). Update on the Impact, Diagnosis and Management of Cardiovascular Autonomic Neuropathy in Diabetes: What Is Defined, What Is New, and What Is Unmet. Diabetes Metab. J..

[73] Ciccacci C., Latini A., Greco C., Politi C., D’Amato C., Lauro D., Novelli G., Borgiani P., Spallone V. (2018). Association between a MIR499A polymorphism and diabetic neuropathy in type 2 diabetes. J. Diabetes Complicat..

[74] Politi C., Ciccacci C., D’Amato C., Novelli G., Borgiani P., Spallone V. (2016). Recent advances in exploring the genetic susceptibility to diabetic neuropathy. Diabetes Res. Clin. Pract..

[75] Kaze A.D., Yuyun M.F., Erqou S., Fonarow G.C., Echouffo-Tcheugui J.B. (2022). Cardiac autonomic neuropathy and risk of incident heart failure among adults with type 2 diabetes. Eur. J. Heart Fail..

[76] Hammer S., van der Meer R.W., Lamb H.J., Schar M., de Roos A., Smit J.W., Romijn J.A. (2008). Progressive caloric restriction induces dose-dependent changes in myocardial triglyceride content and diastolic function in healthy men. J. Clin. Endocrinol. Metab..

[77] Zhang D., Wan A., Chiu A.P., Wang Y., Wang F., Neumaier K., Lal N., Bround M.J., Johnson J.D., Vlodavsky I. (2013). Hyperglycemia-induced secretion of endothelial heparanase stimulates a vascular endothelial growth factor autocrine network in cardiomyocytes that promotes recruitment of lipoprotein lipase. Arterioscler. Thromb. Vasc. Biol..

[78] Schulze P.C., Drosatos K., Goldberg I.J. (2016). Lipid use and misuse by the heart. Circ. Res..

[79] Mahmod M., Pal N., Rayner J., Holloway C., Raman B., Dass S., Levelt E., Ariga R., Ferreira V., Banerjee R. (2018). The interplay between metabolic alterations, diastolic strain rate and exercise capacity in mild heart failure with preserved ejection fraction: A cardiovascular magnetic resonance study. J. Cardiovasc. Magn. Reson..

[80] Wei J., Nelson M.D., Szczepaniak E.W., Smith L., Mehta P.K., Thomson L.E., Berman D.S., Li D., Bairey Merz C.N., Szczepaniak L.S. (2016). Myocardial steatosis as a possible mechanistic link between diastolic dysfunction and coronary microvascular dysfunction in women. Am. J. Physiol. Heart Circ. Physiol..

[81] Wu C.K., Lee J.K., Hsu J.C., Su M.M., Wu Y.F., Lin T.T., Lan C.W., Hwang J.J., Lin L.Y. (2020). Myocardial adipose deposition and the development of heart failure with preserved ejection fraction. Eur. J. Heart Fail..

[82] McGavock J.M., Lingvay I., Zib I., Tillery T., Salas N., Unger R., Levine B.D., Raskin P., Victor R.G., Szczepaniak L.S. (2007). Cardiac steatosis in diabetes mellitus: A 1H-magnetic resonance spectroscopy study. Circulation.

[83] Bakris G.L., Fonseca V.A., Sharma K., Wright E.M. (2009). Renal sodium-glucose transport: Role in diabetes mellitus and potential clinical implications. Kidney Int..

[84] Lee Y.J., Lee Y.J., Han H.J. (2007). Regulatory mechanisms of Na(+)/glucose cotransporters in renal proximal tubule cells. Kidney Int..

[85] Rossetti L., Smith D., Shulman G.I., Papachristou D., DeFronzo R.A. (1987). Correction of hyperglycemia with phlorizin normalizes tissue sensitivity to insulin in diabetic rats. J. Clin. Investig..

[86] Meng W., Ellsworth B.A., Nirschl A.A., McCann P.J., Patel M., Girotra R.N., Wu G., Sher P.M., Morrison E.P., Biller S.A. (2008). Discovery of dapagliflozin: A potent, selective renal sodium-dependent glucose cotransporter 2 (SGLT2) inhibitor for the treatment of type 2 diabetes. J. Med. Chem..

[87] Nomura S., Sakamaki S., Hongu M., Kawanishi E., Koga Y., Sakamoto T., Yamamoto Y., Ueta K., Kimata H., Nakayama K. (2010). Discovery of canagliflozin, a novel C-glucoside with thiophene ring, as sodium-dependent glucose cotransporter 2 inhibitor for the treatment of type 2 diabetes mellitus. J. Med. Chem..

[88] Grempler R., Thomas L., Eckhardt M., Himmelsbach F., Sauer A., Sharp D.E., Bakker R.A., Mark M., Klein T., Eickelmann P. (2012). Empagliflozin, a novel selective sodium glucose cotransporter-2 (SGLT-2) inhibitor: Characterisation and comparison with other SGLT-2 inhibitors. Diabetes Obes. Metab..

[89] Mascitti V., Maurer T.S., Robinson R.P., Bian J., Boustany-Kari C.M., Brandt T., Collman B.M., Kalgutkar A.S., Klenotic M.K., Leininger M.T. (2011). Discovery of a clinical candidate from the structurally unique dioxa-bicyclo[3.2.1]octane class of sodium-dependent glucose cotransporter 2 inhibitors. J. Med. Chem..

[90] Yasui A., Lee G., Hirase T., Kaneko T., Kaspers S., von Eynatten M., Okamura T. (2018). Empagliflozin Induces Transient Diuresis Without Changing Long-Term Overall Fluid Balance in Japanese Patients With Type 2 Diabetes. Diabetes Ther..

[91] Damman K., Beusekamp J.C., Boorsma E.M., Swart H.P., Smilde T.D.J., Elvan A., van Eck J.W.M., Heerspink H.J.L., Voors A.A. (2020). Randomized, double-blind, placebo-controlled, multicentre pilot study on the effects of empagliflozin on clinical outcomes in patients with acute decompensated heart failure (EMPA-RESPONSE-AHF). Eur. J. Heart Fail..

[92] Nassif M.E., Windsor S.L., Tang F., Khariton Y., Husain M., Inzucchi S.E., McGuire D.K., Pitt B., Scirica B.M., Austin B. (2019). Dapagliflozin Effects on Biomarkers, Symptoms, and Functional Status in Patients With Heart Failure With Reduced Ejection Fraction: The DEFINE-HF Trial. Circulation.

[93] Theofilis P., Antonopoulos A.S., Katsimichas T., Oikonomou E., Siasos G., Aggeli C., Tsioufis K., Tousoulis D. (2022). The impact of SGLT2 inhibition on imaging markers of cardiac function: A systematic review and meta-analysis. Pharmacol. Res..

[94] Ren F.F., Xie Z.Y., Jiang Y.N., Guan X., Chen Q.Y., Lai T.F., Li L. (2022). Dapagliflozin attenuates pressure overload-induced myocardial remodeling in mice via activating SIRT1 and inhibiting endoplasmic reticulum stress. Acta Pharmacol. Sin..

[95] Yu Y.W., Que J.Q., Liu S., Huang K.Y., Qian L., Weng Y.B., Rong F.N., Wang L., Zhou Y.Y., Xue Y.J. (2021). Sodium-Glucose Co-transporter-2 Inhibitor of Dapagliflozin Attenuates Myocardial Ischemia/Reperfusion Injury by Limiting NLRP3 Inflammasome Activation and Modulating Autophagy. Front. Cardiovasc. Med..

[96] Quagliariello V., De Laurentiis M., Rea D., Barbieri A., Monti M.G., Carbone A., Paccone A., Altucci L., Conte M., Canale M.L. (2021). The SGLT-2 inhibitor empagliflozin improves myocardial strain, reduces cardiac fibrosis and pro-inflammatory cytokines in non-diabetic mice treated with doxorubicin. Cardiovasc. Diabetol..

[97] Sukhanov S., Higashi Y., Yoshida T., Mummidi S., Aroor A.R., Jeffrey Russell J., Bender S.B., DeMarco V.G., Chandrasekar B. (2021). The SGLT2 inhibitor Empagliflozin attenuates interleukin-17A-induced human aortic smooth muscle cell proliferation and migration by targeting TRAF3IP2/ROS/NLRP3/Caspase-1-dependent IL-1beta and IL-18 secretion. Cell Signal.

[98] Zhang N., Feng B., Ma X., Sun K., Xu G., Zhou Y. (2019). Dapagliflozin improves left ventricular remodeling and aorta sympathetic tone in a pig model of heart failure with preserved ejection fraction. Cardiovasc. Diabetol..

[99] Kondo H., Akoumianakis I., Badi I., Akawi N., Kotanidis C.P., Polkinghorne M., Stadiotti I., Sommariva E., Antonopoulos A.S., Carena M.C. (2021). Effects of canagliflozin on human myocardial redox signalling: Clinical implications. Eur. Heart J..

[100] Cappetta D., De Angelis A., Ciuffreda L.P., Coppini R., Cozzolino A., Micciche A., Dell’Aversana C., D’Amario D., Cianflone E., Scavone C. (2020). Amelioration of diastolic dysfunction by dapagliflozin in a non-diabetic model involves coronary endothelium. Pharmacol. Res..

[101] Madonna R., Doria V., Minnucci I., Pucci A., Pierdomenico D.S., De Caterina R. (2020). Empagliflozin reduces the senescence of cardiac stromal cells and improves cardiac function in a murine model of diabetes. J. Cell Mol. Med..

[102] Young S.L., Ryan L., Mullins T.P., Flint M., Steane S.E., Walton S.L., Bielefeldt-Ohmann H., Carter D.A., Reichelt M.E., Gallo L.A. (2021). Sotagliflozin, a Dual SGLT1/2 Inhibitor, Improves Cardiac Outcomes in a Normoglycemic Mouse Model of Cardiac Pressure Overload. Front. Physiol..

[103] Tian J., Zhang M., Suo M., Liu D., Wang X., Liu M., Pan J., Jin T., An F. (2021). Dapagliflozin alleviates cardiac fibrosis through suppressing EndMT and fibroblast activation via AMPKα/TGF-β/Smad signalling in type 2 diabetic rats. J. Cell Mol. Med..

[104] Zhang Y., Lin X., Chu Y., Chen X., Du H., Zhang H., Xu C., Xie H., Ruan Q., Lin J. (2021). Dapagliflozin: A sodium-glucose cotransporter 2 inhibitor, attenuates angiotensin II-induced cardiac fibrotic remodeling by regulating TGFβ1/Smad signaling. Cardiovasc. Diabetol..

[105] Liu Y., Wu M., Xu J., Xu B., Kang L. (2021). Empagliflozin prevents from early cardiac injury post myocardial infarction in non-diabetic mice. Eur. J. Pharm. Sci..

[106] Santos-Gallego C.G., Requena-Ibanez J.A., San Antonio R., Garcia-Ropero A., Ishikawa K., Watanabe S., Picatoste B., Vargas-Delgado A.P., Flores-Umanzor E.J., Sanz J. (2021). Empagliflozin Ameliorates Diastolic Dysfunction and Left Ventricular Fibrosis/Stiffness in Nondiabetic Heart Failure: A Multimodality Study. JACC Cardiovasc. Imaging.

[107] Marfella R., D’Onofrio N., Trotta M.C., Sardu C., Scisciola L., Amarelli C., Balestrieri M.L., Grimaldi V., Mansueto G., Esposito S. (2022). Sodium/glucose cotransporter 2 (SGLT2) inhibitors improve cardiac function by reducing JunD expression in human diabetic hearts. Metabolism.

[108] Sun P., Wang Y., Ding Y., Luo J., Zhong J., Xu N., Zhang Y., Xie W. (2021). Canagliflozin attenuates lipotoxicity in cardiomyocytes and protects diabetic mouse hearts by inhibiting the mTOR/HIF-1alpha pathway. iScience.

[109] Di Franco A., Cantini G., Tani A., Coppini R., Zecchi-Orlandini S., Raimondi L., Luconi M., Mannucci E. (2017). Sodium-dependent glucose transporters (SGLT) in human ischemic heart: A new potential pharmacological target. Int. J. Cardiol..

[110] Theofilis P., Sagris M., Oikonomou E., Antonopoulos A.S., Siasos G., Tsioufis K., Tousoulis D. (2022). Pleiotropic effects of SGLT2 inhibitors and heart failure outcomes. Diabetes Res. Clin. Pract..

[111] Inzucchi S.E., Zinman B., Fitchett D., Wanner C., Ferrannini E., Schumacher M., Schmoor C., Ohneberg K., Johansen O.E., George J.T. (2018). How Does Empagliflozin Reduce Cardiovascular Mortality? Insights From a Mediation Analysis of the EMPA-REG OUTCOME Trial. Diabetes Care.

[112] Li J., Woodward M., Perkovic V., Figtree G.A., Heerspink H.J.L., Mahaffey K.W., de Zeeuw D., Vercruysse F., Shaw W., Matthews D.R. (2020). Mediators of the Effects of Canagliflozin on Heart Failure in Patients With Type 2 Diabetes. JACC Heart Fail..

[113] Mazer C.D., Hare G.M.T., Connelly P.W., Gilbert R.E., Shehata N., Quan A., Teoh H., Leiter L.A., Zinman B., Juni P. (2020). Effect of Empagliflozin on Erythropoietin Levels, Iron Stores, and Red Blood Cell Morphology in Patients With Type 2 Diabetes Mellitus and Coronary Artery Disease. Circulation.

[114] Solak Y., Cetiner M., Siriopol D., Tarim K., Afsar B., Covic A., Kanbay M. (2016). Novel Masters of Erythropoiesis: Hypoxia Inducible Factors and Recent Advances in Anemia of Renal Disease. Blood Purif.

[115] Swedberg K., Young J.B., Anand I.S., Cheng S., Desai A.S., Diaz R., Maggioni A.P., McMurray J.J., O’Connor C., Pfeffer M.A. (2013). Treatment of anemia with darbepoetin alfa in systolic heart failure. N. Engl. J. Med..

[116] Jiang K., Xu Y., Wang D., Chen F., Tu Z., Qian J., Xu S., Xu Y., Hwa J., Li J. (2022). Cardioprotective mechanism of SGLT2 inhibitor against myocardial infarction is through reduction of autosis. Protein Cell.

[117] Wang C.C., Li Y., Qian X.Q., Zhao H., Wang D., Zuo G.X., Wang K. (2022). Empagliflozin alleviates myocardial I/R injury and cardiomyocyte apoptosis via inhibiting ER stress-induced autophagy and the PERK/ATF4/Beclin1 pathway. J. Drug Target.

[118] Zannad F., Ferreira J.P., Butler J., Filippatos G., Januzzi J.L., Sumin M., Zwick M., Saadati M., Pocock S.J., Sattar N. (2022). Effect of empagliflozin on circulating proteomics in heart failure: Mechanistic insights into the EMPEROR programme. Eur. Heart J..

[119] Theofilis P., Vordoni A., Kalaitzidis R.G. (2022). Oxidative Stress Management in Cardiorenal Diseases: Focus on Novel Antidiabetic Agents, Finerenone, and Melatonin. Life.

[120] Theofilis P., Sagris M., Oikonomou E., Antonopoulos A.S., Siasos G., Tsioufis K., Tousoulis D. (2022). The Anti-Inflammatory Effect of Novel Antidiabetic Agents. Life.

[121] Theofilis P., Sagris M., Oikonomou E., Antonopoulos A.S., Siasos G., Tsioufis K., Tousoulis D. (2022). The impact of SGLT2 inhibitors on inflammation: A systematic review and meta-analysis of studies in rodents. Int. Immunopharmacol..

[122] Bray J.J.H., Foster-Davies H., Stephens J.W. (2020). A systematic review examining the effects of sodium-glucose cotransporter-2 inhibitors (SGLT2is) on biomarkers of inflammation and oxidative stress. Diabetes Res. Clin. Pract..

[123] Sardu C., Massetti M., Testa N., Martino L.D., Castellano G., Turriziani F., Sasso F.C., Torella M., De Feo M., Santulli G. (2021). Effects of Sodium-Glucose Transporter 2 Inhibitors (SGLT2-I) in Patients With Ischemic Heart Disease (IHD) Treated by Coronary Artery Bypass Grafting via MiECC: Inflammatory Burden, and Clinical Outcomes at 5 Years of Follow-Up. Front. Pharmacol..

[124] Sposito A.C., Breder I., Soares A.A.S., Kimura-Medorima S.T., Munhoz D.B., Cintra R.M.R., Bonilha I., Oliveira D.C., Breder J.C., Cavalcante P. (2021). Dapagliflozin effect on endothelial dysfunction in diabetic patients with atherosclerotic disease: A randomized active-controlled trial. Cardiovasc. Diabetol..

[125] Eriksson J.W., Lundkvist P., Jansson P.A., Johansson L., Kvarnstrom M., Moris L., Miliotis T., Forsberg G.B., Riserus U., Lind L. (2018). Effects of dapagliflozin and n-3 carboxylic acids on non-alcoholic fatty liver disease in people with type 2 diabetes: A double-blind randomised placebo-controlled study. Diabetologia.

[126] Bosch A., Ott C., Jung S., Striepe K., Karg M.V., Kannenkeril D., Dienemann T., Schmieder R.E. (2019). How does empagliflozin improve arterial stiffness in patients with type 2 diabetes mellitus? Sub analysis of a clinical trial. Cardiovasc. Diabetol..

[127] Bouchi R., Terashima M., Sasahara Y., Asakawa M., Fukuda T., Takeuchi T., Nakano Y., Murakami M., Minami I., Izumiyama H. (2017). Luseogliflozin reduces epicardial fat accumulation in patients with type 2 diabetes: A pilot study. Cardiovasc. Diabetol..

[128] Requena-Ibanez J.A., Santos-Gallego C.G., Rodriguez-Cordero A., Vargas-Delgado A.P., Mancini D., Sartori S., Atallah-Lajam F., Giannarelli C., Macaluso F., Lala A. (2021). Mechanistic Insights of Empagliflozin in Nondiabetic Patients With HFrEF: From the EMPA-TROPISM Study. JACC Heart Fail..

[129] Wang D., Liu J., Zhong L., Li S., Zhou L., Zhang Q., Li M., Xiao X. (2022). The effect of sodium-glucose cotransporter 2 inhibitors on biomarkers of inflammation: A systematic review and meta-analysis of randomized controlled trials. Front. Pharmacol..

[130] Lin Y.-W., Chen C.-Y., Shih J.-Y., Cheng B.-C., Chang C.-P., Lin M.-T., Ho C.-H., Chen Z.-C., Fisch S., Chang W.-T. (2021). Dapagliflozin Improves Cardiac Hemodynamics and Mitigates Arrhythmogenesis in Mitral Regurgitation-Induced Myocardial Dysfunction. J. Am. Heart Assoc..

[131] Hsu J.-C., Wang C.-Y., Su M.-Y.M., Lin L.-Y., Yang W.-S. (2019). Effect of Empagliflozin on Cardiac Function, Adiposity, and Diffuse Fibrosis in Patients with Type 2 Diabetes Mellitus. Sci. Rep..

[132] Mason T., Coelho-Filho O.R., Verma S., Chowdhury B., Zuo F., Quan A., Thorpe K.E., Bonneau C., Teoh H., Gilbert R.E. (2021). Empagliflozin Reduces Myocardial Extracellular Volume in Patients With Type 2 Diabetes and Coronary Artery Disease. JACC Cardiovasc. Imaging.

[133] Packer M. (2021). Differential Pathophysiological Mechanisms in Heart Failure With a Reduced or Preserved Ejection Fraction in Diabetes. JACC Heart Fail..

[134] Salvatore T., Galiero R., Caturano A., Vetrano E., Rinaldi L., Coviello F., Di Martino A., Albanese G., Colantuoni S., Medicamento G. (2022). Dysregulated Epicardial Adipose Tissue as a Risk Factor and Potential Therapeutic Target of Heart Failure with Preserved Ejection Fraction in Diabetes. Biomolecules.

[135] Diaz-Rodriguez E., Agra R.M., Fernandez A.L., Adrio B., Garcia-Caballero T., Gonzalez-Juanatey J.R., Eiras S. (2018). Effects of dapagliflozin on human epicardial adipose tissue: Modulation of insulin resistance, inflammatory chemokine production, and differentiation ability. Cardiovasc. Res..

[136] Trum M., Riechel J., Lebek S., Pabel S., Sossalla S.T., Hirt S., Arzt M., Maier L.S., Wagner S. (2020). Empagliflozin inhibits Na(+) /H(+) exchanger activity in human atrial cardiomyocytes. ESC Heart Fail..

[137] Zuurbier C.J., Baartscheer A., Schumacher C.A., Fiolet J.W.T., Coronel R. (2021). SGLT2 inhibitor empagliflozin inhibits the cardiac Na+/H+ exchanger 1: Persistent inhibition under various experimental conditions. Cardiovasc. Res..

[138] Chung Y.J., Park K.C., Tokar S., Eykyn T.R., Fuller W., Pavlovic D., Swietach P., Shattock M.J. (2021). SGLT2 inhibitors and the cardiac Na+/H+ exchanger-1: The plot thickens. Cardiovasc. Res..

[139] Gaborit B., Ancel P., Abdullah A.E., Maurice F., Abdesselam I., Calen A., Soghomonian A., Houssays M., Varlet I., Eisinger M. (2021). Effect of empagliflozin on ectopic fat stores and myocardial energetics in type 2 diabetes: The EMPACEF study. Cardiovasc. Diabetol..

[140] Sardu C., Massimo Massetti M., Rambaldi P., Gatta G., Cappabianca S., Sasso F.C., Santamaria M., Volpicelli M., Ducceschi V., Signoriello G. (2022). SGLT2-inhibitors reduce the cardiac autonomic neuropathy dysfunction and vaso-vagal syncope recurrence in patients with type 2 diabetes mellitus: The SCAN study. Metabolism.

[141] Balcioglu A.S., Celik E., Sahin M., Gocer K., Aksu E., Aykan A.C. (2022). Dapagliflozin Improves Cardiac Autonomic Function Measures in Type 2 Diabetic Patients with Cardiac Autonomic Neuropathy. Anatol. J. Cardiol..

[142] Patoulias D., Katsimardou A., Fragakis N., Papadopoulos C., Doumas M. (2023). Effect of SGLT-2 inhibitors on cardiac autonomic function in type 2 diabetes mellitus: A meta-analysis of randomized controlled trials. Acta Diabetol..

[143] Hamaoka T., Murai H., Hirai T., Sugimoto H., Mukai Y., Inoue O., Takashima S., Kato T., Takata S., Usui S. (2021). Different Responses of Muscle Sympathetic Nerve Activity to Dapagliflozin Between Patients With Type 2 Diabetes With and Without Heart Failure. J. Am. Heart Assoc..

[144] Theofilis P., Kalaitzidis R.G. (2022). SGLT2 inhibitors and kidney diseases: A clinical perspective. Curr. Med. Chem..

[145] McMurray J.J.V., Solomon S.D., Inzucchi S.E., Kober L., Kosiborod M.N., Martinez F.A., Ponikowski P., Sabatine M.S., Anand I.S., Belohlavek J. (2019). Dapagliflozin in Patients with Heart Failure and Reduced Ejection Fraction. N. Engl. J. Med..

[146] Butt J.H., Nicolau J.C., Verma S., Docherty K.F., Petrie M.C., Inzucchi S.E., Schou M., Kosiborod M.N., Langkilde A.M., Martinez F.A. (2021). Efficacy and safety of dapagliflozin according to aetiology in heart failure with reduced ejection fraction: Insights from the DAPA-HF trial. Eur. J. Heart Fail.

[147] Solomon S.D., Jhund P.S., Claggett B.L., Dewan P., Kober L., Kosiborod M.N., Martinez F.A., Ponikowski P., Sabatine M.S., Inzucchi S.E. (2020). Effect of Dapagliflozin in Patients With HFrEF Treated With Sacubitril/Valsartan: The DAPA-HF Trial. JACC Heart Fail..

[148] Kosiborod M.N., Jhund P.S., Docherty K.F., Diez M., Petrie M.C., Verma S., Nicolau J.C., Merkely B., Kitakaze M., DeMets D.L. (2020). Effects of Dapagliflozin on Symptoms, Function, and Quality of Life in Patients With Heart Failure and Reduced Ejection Fraction: Results From the DAPA-HF Trial. Circulation.

[149] Yeoh S.E., Dewan P., Jhund P.S., Inzucchi S.E., Kober L., Kosiborod M.N., Martinez F.A., Ponikowski P., Sabatine M.S., Solomon S.D. (2020). Patient Characteristics, Clinical Outcomes, and Effect of Dapagliflozin in Relation to Duration of Heart Failure: Is It Ever Too Late to Start a New Therapy?. Circ. Heart Fail..

[150] Dewan P., Solomon S.D., Jhund P.S., Inzucchi S.E., Kober L., Kosiborod M.N., Martinez F.A., Ponikowski P., DeMets D.L., Sabatine M.S. (2020). Efficacy and safety of sodium-glucose co-transporter 2 inhibition according to left ventricular ejection fraction in DAPA-HF. Eur. J. Heart Fail..

[151] Packer M., Anker S.D., Butler J., Filippatos G., Pocock S.J., Carson P., Januzzi J., Verma S., Tsutsui H., Brueckmann M. (2020). Cardiovascular and Renal Outcomes with Empagliflozin in Heart Failure. N. Engl. J. Med..

[152] Packer M., Anker S.D., Butler J., Filippatos G., Ferreira J.P., Pocock S.J., Rocca H.B., Janssens S., Tsutsui H., Zhang J. (2021). Influence of neprilysin inhibition on the efficacy and safety of empagliflozin in patients with chronic heart failure and a reduced ejection fraction: The EMPEROR-Reduced trial. Eur. Heart J..

[153] Zannad F., Ferreira J.P., Pocock S.J., Zeller C., Anker S.D., Butler J., Filippatos G., Hauske S.J., Brueckmann M., Pfarr E. (2021). Cardiac and Kidney Benefits of Empagliflozin in Heart Failure Across the Spectrum of Kidney Function: Insights From EMPEROR-Reduced. Circulation.

[154] Anker S.D., Butler J., Filippatos G., Khan M.S., Marx N., Lam C.S.P., Schnaidt S., Ofstad A.P., Brueckmann M., Jamal W. (2021). Effect of Empagliflozin on Cardiovascular and Renal Outcomes in Patients With Heart Failure by Baseline Diabetes Status: Results From the EMPEROR-Reduced Trial. Circulation.

[155] Bhatt D.L., Szarek M., Steg P.G., Cannon C.P., Leiter L.A., McGuire D.K., Lewis J.B., Riddle M.C., Voors A.A., Metra M. (2021). Sotagliflozin in Patients with Diabetes and Recent Worsening Heart Failure. N. Engl. J. Med..

[156] Anker S.D., Butler J., Filippatos G., Ferreira J.P., Bocchi E., Bohm M., Brunner-La Rocca H.P., Choi D.J., Chopra V., Chuquiure-Valenzuela E. (2021). Empagliflozin in Heart Failure with a Preserved Ejection Fraction. N. Engl. J. Med..

[157] Solomon S.D., McMurray J.J.V., Claggett B., de Boer R.A., DeMets D., Hernandez A.F., Inzucchi S.E., Kosiborod M.N., Lam C.S.P., Martinez F. (2022). Dapagliflozin in Heart Failure with Mildly Reduced or Preserved Ejection Fraction. N. Engl. J. Med..

[158] Vaduganathan M., Claggett B.L., Jhund P., de Boer R.A., Hernandez A.F., Inzucchi S.E., Kosiborod M.N., Lam C.S.P., Martinez F., Shah S.J. (2022). Time to Clinical Benefit of Dapagliflozin in Patients With Heart Failure With Mildly Reduced or Preserved Ejection Fraction: A Prespecified Secondary Analysis of the DELIVER Randomized Clinical Trial. JAMA Cardiol..

[159] Kosiborod M.N., Bhatt A.S., Claggett B.L., Vaduganathan M., Kulac I.J., Lam C.S.P., Hernandez A.F., Martinez F.A., Inzucchi S.E., Shah S.J. (2022). Effect of Dapagliflozin on Health Status in Patients With Preserved or Mildly Reduced Ejection Fraction. J. Am. Coll Cardiol..

[160] Butt J.H., Jhund P.S., Belohlavek J., de Boer R.A., Chiang C.E., Desai A.S., Drozdz J., Hernandez A.F., Inzucchi S.E., Katova T. (2022). Efficacy and Safety of Dapagliflozin According to Frailty in Patients With Heart Failure: A Prespecified Analysis of the DELIVER Trial. Circulation.

[161] Adamson C., Kondo T., Jhund P.S., de Boer R.A., Cabrera Honorio J.W., Claggett B., Desai A.S., Alcocer Gamba M.A., Al Habeeb W., Hernandez A.F. (2022). Dapagliflozin for heart failure according to body mass index: The DELIVER trial. Eur. Heart J..

[162] Peikert A., Martinez F.A., Vaduganathan M., Claggett B.L., Kulac I.J., Desai A.S., Jhund P.S., de Boer R.A., DeMets D., Hernandez A.F. (2022). Efficacy and Safety of Dapagliflozin in Heart Failure With Mildly Reduced or Preserved Ejection Fraction According to Age: The DELIVER Trial. Circ. Heart Fail..

[163] Yang M., Butt J.H., Kondo T., Jering K.S., Docherty K.F., Jhund P.S., de Boer R.A., Claggett B.L., Desai A.S., Hernandez A.F. (2022). Dapagliflozin in patients with heart failure with mildly reduced and preserved ejection fraction treated with a mineralocorticoid receptor antagonist or sacubitril/valsartan. Eur. J. Heart Fail..

[164] Inzucchi S.E., Claggett B.L., Vaduganathan M., Desai A.S., Jhund P.S., de Boer R.A., Hernandez A.F., Kosiborod M.N., Lam C.S.P., Martinez F. (2022). Efficacy and safety of dapagliflozin in patients with heart failure with mildly reduced or preserved ejection fraction by baseline glycaemic status (DELIVER): A subgroup analysis from an international, multicentre, double-blind, randomised, placebo-controlled trial. Lancet Diabetes Endocrinol..

[165] Talha K.M., Butler J., Greene S.J., Aggarwal R., Anker S.D., Claggett B.L., Solomon S.D., McMurray J.J.V., Vaduganathan M., Fonarow G.C. (2022). Population-Level Implications of Sodium-Glucose Cotransporter-2 Inhibitors for Heart Failure With Preserved Ejection Fraction in the US. JAMA Cardiol..

